# The Two-Hand Approach to Retrograde Intubation

**DOI:** 10.7759/cureus.95405

**Published:** 2025-10-25

**Authors:** Armaan Chokshi, Jyothika Annareddy, Amit Aggarwal, Sharif Mohamed

**Affiliations:** 1 Anesthesiology, University of Texas Medical Branch at Galveston, Galveston, USA

**Keywords:** anesthesia technique, difficult airway algorithm, difficult airway intubation, difficult airway management, novel technique, retrograde intubation

## Abstract

Retrograde intubation is a minimally invasive and underutilized airway rescue technique that bypasses the need for direct visualization of the vocal cords by passing a guidewire from the cricothyroid membrane to the nasopharynx or oropharynx, creating a pathway for an endotracheal tube (ETT) into the trachea. This technique is particularly valuable in difficult airway scenarios such as bleeding, edema, distorted anatomy, maxillofacial trauma, or cervical spine immobility, and utilizes simple equipment and common anatomical landmarks, making it efficient for emergency or resource-limited settings. The standard procedure involves puncturing the cricothyroid membrane with a needle, advancing the guidewire cephalad, guiding an airway exchange catheter over the guidewire, threading the ETT over the catheter, and removing the guidewire and catheter. However, a major challenge is dislodgment of the catheter from its subglottic position. The two-hand modification addresses this problem by having the operator stabilize the ETT at the nares with one hand while securing the airway exchange catheter with the other, allowing controlled movement and minimizing displacement during guidewire and catheter withdrawal. While complications such as bleeding, subcutaneous emphysema, pneumomediastinum, infection, guidewire or catheter kinking, and vocal cord trauma can occur, these risks can be mitigated by using the two-hand modification combined with tools such as suction, video laryngoscopy, and fiberoptic scopes, making retrograde intubation with the two-hand approach a reliable, lifesaving technique that provides improved stabilization, reduced complications, and increased success rates for difficult airway management.

## Introduction

Retrograde intubation is a vital but underutilized rescue airway technique first described in the 1960s that involves puncturing the cricothyroid membrane, passing a guidewire cephalad into the oropharynx or nasopharynx, and passing an endotracheal tube (ETT) into the trachea, bypassing the need for direct visualization of the vocal cords [1,2]. This technique offers unique advantages, particularly in scenarios where there is heavy bleeding, airway swelling, distorted anatomy, or poor airway visualization, where advanced equipment, such as a flexible bronchoscope, may be unavailable. This intubation technique relies on simple equipment and the cricothyroid membrane landmark, making it ideal for resource-limited and emergency settings [1-3]. Retrograde intubation remains clinically effective in "cannot intubate, cannot oxygenate" scenarios when the patient is hemodynamically stable, serving as a viable intermediate option after conventional techniques, such as bag-mask ventilation, and supraglottic airways have failed but before committing to surgical cricothyroidotomy [1], which carries higher risks of complications such as hemorrhage, subglottic stenosis, esophageal injury, and ETT misplacement [4]. Retrograde intubation, by contrast, is minimally invasive and provides an intermediate step that can avoid the morbidity associated with emergent surgical airways. Ultimately, as time permits with stable hemodynamics, retrograde intubation should be strongly considered prior to committing to a surgical airway.

## Technical report

Indications and benefits

Retrograde intubation is an important and underutilized technique in airway management [1]. By relying on anatomical landmarks rather than direct visualization, it offers a reliable alternative for securing the airway in difficult scenarios. The procedure is especially useful in settings of poor glottis visualization, maxillofacial trauma, restricted cervical spine mobility, limited mouth opening, or two or more consecutive failed intubation attempts, where other techniques may prove to be inadequate or unsafe [2,5,6]. This section will explore the clinical indications for retrograde intubation and highlight its key benefits.

Retrograde intubation is particularly valuable in patients with difficult airways where visualization of the vocal cords is limited or impossible. In patients with high Cormack-Lehane grades (III or IV), standard direct or video laryngoscopy may fail to provide an adequate view of the glottis, in which case other non-invasive techniques such as hyperangulated video laryngoscopy, flexible scope intubation, and the use of jet ventilation are typically attempted [7]. If these approaches fail, then it is important to consider more invasive options, including retrograde intubation. Retrograde intubation has the key benefit of bypassing the need for visual confirmation of vocal cord anatomy by allowing for blind passage of the ETT guided by a wire introduced through the cricothyroid membrane [2,7]. This technique can facilitate successful intubation while avoiding multiple attempts that could worsen airway edema or bleeding [5].

Additionally, patients with significant maxillofacial trauma or head and neck cancers often present with distorted anatomy, active bleeding, and limited access to oral or nasal airways [6,8]. In such settings, retrograde intubation should be considered, as it avoids passage through the disrupted upper airway structures and allows airway access through a retrograde approach [6,9]. By accessing the trachea through a percutaneous approach and guiding the tube from below, the airway can be secured with minimal disturbance of any facial or maxillofacial injuries, reducing the risk of further trauma [6,9].

 Another indication for retrograde intubation is in trauma patients with cervical spine injury [1,6]. Maintaining cervical spine alignment in these patients during airway management is crucial. Retrograde intubation is an optimal choice for these patients as it requires little to no manipulation of the head and neck, making it a suitable option when manual in-line stabilization or cervical collars are in place [6,7]. Retrograde intubation has also been found to be successful in fewer attempts, preventing the number of traumatic attempts to secure an airway [5].

Technique description

Standard Retrograde Intubation Technique

Retrograde intubation is an established, albeit infrequently used, airway management technique that serves as a valuable alternative in situations where conventional orotracheal intubation is difficult or unsuccessful. Originally described by otolaryngologists MacLean and Card in 1960 for facilitating airway access during head and neck cancer surgery, the technique has since undergone several modifications to improve its safety and applicability [8]. In 1963, Waters introduced a standardized approach involving puncture of the cricothyroid membrane and cephalad passage of a guidewire to facilitate ETT placement [10].

The standard retrograde intubation technique begins with appropriate patient positioning. The patient is typically placed in a supine position with the neck extended, provided that cervical spine precautions are not required. Para-oxygenation with bag-mask ventilation, or in rare cases non-invasive positive pressure ventilation, should be used in all retrograde intubation attempts. In awake patients, the procedure is performed under local anesthesia with or without sedation; topical anesthesia may also be applied to the oropharynx in these cases to improve patient comfort [10]. Less commonly, retrograde intubation is performed under general anesthesia, typically in patients with an unsuspected difficult laryngoscopy who are already induced and have had multiple previous failed intubation attempts.

The cricothyroid membrane is then identified by palpation, stabilized, and punctured using a sterile 16- or 18-gauge introducer needle attached to a syringe. Entry into the tracheal lumen is confirmed by aspiration of air. The syringe is then removed, and a flexible guidewire, such as an intubation catheter provided in a retrograde intubation kit or a J-tipped guidewire used in ureteroscopic procedures, is advanced through the needle into the trachea and directed cephalad [10]. Once the guidewire exits the mouth or nose, it is advanced a few centimeters further until the positioning marker is at the skin. The introducer needle is then removed, and a hemostat is often used to hold the guidewire in place.

Once the guidewire is visible in the upper airway, it is tensioned to maintain a direct path into the trachea. Most commonly, an airway exchange catheter is threaded over the guidewire, and an ETT is then threaded over the airway exchange catheter and advanced into the trachea. The guidewire is then withdrawn. Then, the ETT is threaded deeper over the airway exchange catheter, until it is subglottic. Resistance may be encountered at the level of the glottis, as the ETT may become impinged on the vocal cords or arytenoid cartilage; gentle manipulation or rotation of the tube may help overcome this. Once the ETT is appropriately positioned, the catheter is withdrawn. Proper placement of the ETT is confirmed by end-tidal carbon dioxide detection, bilateral auscultation, and, when available, fiberoptic bronchoscopy [10-12].

Two-Hand Technique Modification

We describe a novel modification to the standard retrograde intubation technique, specifically applicable when the guidewire or airway exchange catheter exits through the nasal passage. This approach enhances control and stability during ETT advancement, particularly in settings with limited visualization.

**Figure 1:**
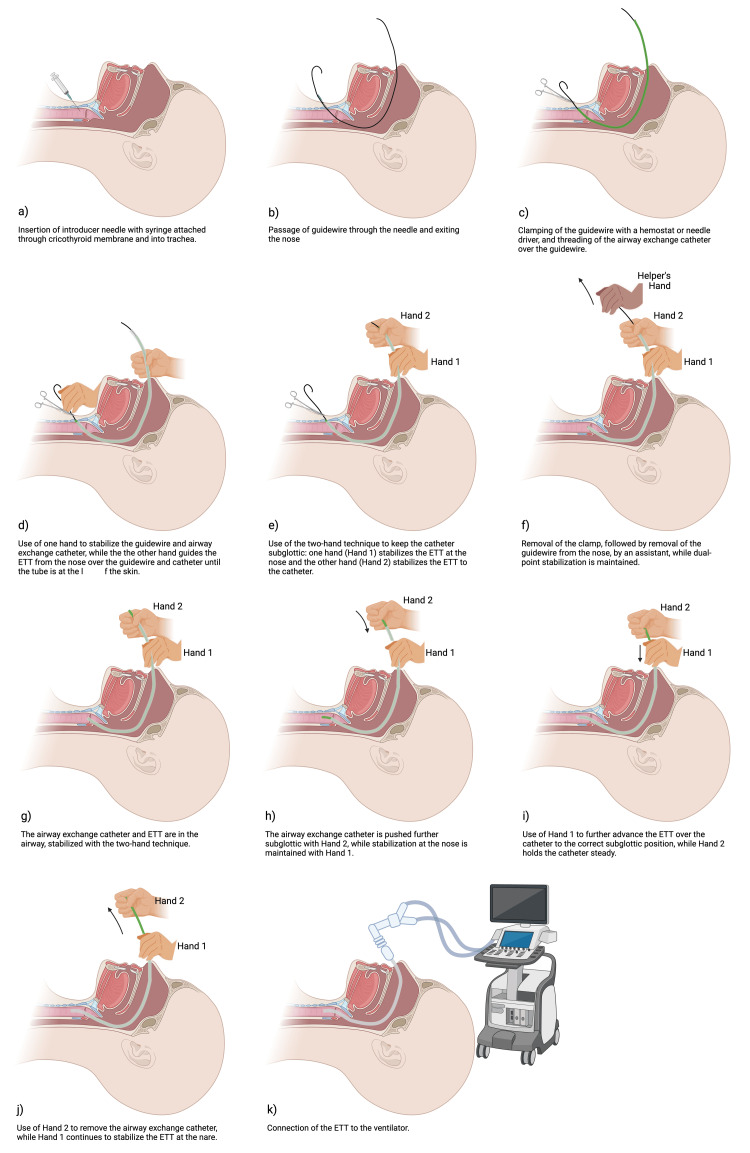
Steps of retrograde intubation using the two-hand technique modification. Image created in BioRender (https://BioRender.com/q7ogcnj) ETT, endotracheal tube

The steps of retrograde intubation using the two-hand technique are illustrated in Figure 1, with the implementation of the two-hand technique beginning in Figure 1e. Once the ETT is advanced from the nose over the guidewire and catheter until it stops, indicating that the tip of the ETT is now subglottic, the operator - positioned at the patient’s head - employs a two-hand stabilization technique to maintain the position of the catheter and ETT tip in the subglottic position. With one hand (termed “Hand 1”), the operator secures the ETT to the nares using a pinching grip: the thumb and index finger grasp the ETT, while the remaining fingers stabilize the nare to prevent backward displacement or unintended movement; the operator then uses their other hand (termed “Hand 2”) to secure the ETT to the airway exchange catheter at a location more distal from the nare (Figure 1e). Following this stabilization, an assisting member from the care team will remove the hemostat or needle driver and then remove the guidewire from the nose, while the operator maintains dual-point stabilization with both hands (Figure 1f). Next, the operator releases Hand 2 and pushes the airway exchange catheter further into the subglottic space with Hand 2, while maintaining stabilization at the nare with Hand 1 (Figure 1h). Then, while holding the catheter steady with Hand 2, the operator releases Hand 1 and uses Hand 1 to further advance the breathing tube over the catheter (Figure 1i). The breathing tube should now be in the correct subglottic position, and the catheter is removed using Hand 2 (Figure 1j). Lastly, the ETT is connected to the ventilator to verify end-tidal carbon dioxide (Figure 1k).

## Discussion

The two-hand technique offers several advantages: it helps maintain the subglottic position of the catheter tip and the ETT, minimizes the risk of accidental ETT misplacement or rotation, and increases operator control with limited direct visualization. Similar to standard retrograde intubation, this modification can also be integrated with adjunctive techniques such as fiberoptic scopes or video laryngoscopy to further optimize airway management in difficult cases.

Complications

Complications of retrograde intubation include bleeding or hematoma formation, subcutaneous emphysema, pneumomediastinum, soft tissue infection, vocal cord trauma, creation of a false passage, misplacement or kinking of the guidewire or ETT, and failure to secure the airway [13]. Bleeding risks are often due to cricothyroid artery injury and may be reduced using a subcricoid approach to tracheal puncture [14]. Subcutaneous emphysema and pneumomediastinum are rare but can result from tissue disruption; limiting the number of needle passes and using an appropriately sized (16- or 18-gauge) needle help prevent these complications [13,15].

Vocal cord trauma can occur if the ETT is blindly advanced after premature guidewire removal, leading to resistance at the glottis or airway distortion [2]. Maintaining the guidewire position during tube passage or using an airway exchange catheter are essential, and adjuncts such as light-guided techniques [16] or flexible fiberoptic bronchoscopy [11,15] can further improve precision and safety by allowing for visualization of the guidewire exit and ETT trajectory.

Soft tissue infections of the neck may result from withdrawing the guidewire through the anterior puncture site, which can introduce skin flora into deeper structures. This can be prevented by retrieving the guidewire through the mouth or nose [2]. The risks of tissue trauma and creation of a false passage may be reduced using a flexible fiberoptic scope during the guidewire retrieval and threading of the ETT. Misplacement or kinking of the ETT is another concern, particularly if the guidewire trajectory deviates from midline; this risk may be mitigated by gentle tensioning of the guidewire and the use of an antegrade sheath or airway exchange catheter to guide the ETT [7]. Loss of airway may also occur if the guidewire or catheter is inadvertently dislodged or shifted into a supraglottic position during tube passage; this can be prevented using the two-hand technique described in this paper.

Although retrograde intubation is a supported technique in patients with difficult airways and previous failed intubation attempts, failure to secure the airway remains the most common complication of this technique, especially in cases of gross anatomical distortion. Combining the technique with other tools can improve the likelihood of success. For example, in cases with copious blood or secretions, a suction catheter or suction-assisted fiberoptic scope, if available, can be incorporated in the retrograde approach to help facilitate passage of the ETT [17]. Regardless of the technique chosen to secure the airway, confirmation of correct ETT placement and discussion of rescue techniques with the care team are imperative to ensuring success [15,16]. Alongside traditional methods such as auscultation and witnessing bilateral chest rise, modern tools such as capnography, end-tidal carbon dioxide detectors, radiography, syringe aspiration, and self-inflating bulb devices (e.g., Ellick’s bulb) should be employed to verify proper tracheal intubation [18].

## Conclusions

Retrograde intubation remains a lifesaving intubation technique, especially for complex cases in both sedated or conscious patients. This paper proposes a two-hand method modification to the standard retrograde intubation technique that provides improved stabilization and maintenance of ETT positioning. By securing the ETT simultaneously at the nares and against the guidewire, this method helps maintain the subglottic position of the catheter tip, reduces the risk of dislodgement, and improves overall control. Ultimately, retrograde intubation, supported by the two-hand modification, can be a valuable option before resorting to surgical airways in difficult airway management.
